# Early presentation of urinary retention in multiple system atrophy: can the disease begin in the sacral spinal cord?

**DOI:** 10.1007/s00415-019-09597-2

**Published:** 2019-11-12

**Authors:** Jalesh N. Panicker, Sara Simeoni, Yasuo Miki, Amit Batla, Valeria Iodice, Janice L. Holton, Ryuji Sakakibara, Thomas T. Warner

**Affiliations:** 1 Department of Uro-Neurology, The National Hospital for Neurology and Neurosurgery, Queen Square, London, WC1N 3BG UK; 2 UCL Queen Square Institute of Neurology, London, WC1N 3BG UK; 3 Reta Lila Weston Institute of Neurological Studies and Queen Square Brain Bank, UCL Queen Square Institute of Neurology, London, UK; 4 Department of Neuropathology, Institute of Brain Science, Hirosaki University Graduate School of Medicine, Hirosaki, Japan; 5 Department of Neurology, Luton and Dunstable University Hospital, Luton, UK; 6 Autonomics Unit, The National Hospital for Neurology and Neurosurgery, Queen Square, London, WC1N 3BG UK; 7 Neurology, Internal Medicine, Sakura Medical Center, Toho University, Sakura, Japan

**Keywords:** MSA, Urinary retention, Multiple system atrophy, EMG, Sacral cord

## Abstract

Lower urinary tract (LUT) dysfunction presents early in multiple system atrophy (MSA), usually initially as urinary urgency, frequency and incontinence, and voiding difficulties/urinary retention becomes apparent over time. We have observed a subset of patients who instead presented initially with urinary retention requiring catheterisation. At presentation, these patients had only subtle neurological signs that would not fulfil the diagnostic criteria of MSA; however, the anal sphincter electromyography (EMG) was abnormal and they reported bowel and sexual dysfunction, suggesting localisation at the level of the sacral spinal cord. They subsequently developed classical neurological signs, meeting the diagnostic criteria for probable MSA. One patient was confirmed to have MSA at autopsy. We postulate that in a subset of patients with MSA, the disease begins in the sacral spinal cord and then spreads to other regions resulting in the classical signs of MSA. The transmissibility of alpha-synuclein has been demonstrated in animal models and the spread of pathology from sacral cord to other regions of the central nervous system is therefore plausible. Patients presenting with urinary retention and mild neurological features would be an ideal group for experimental trials evaluating neuroprotection in MSA

## Introduction

Multiple system atrophy (MSA) is a progressive neurodegenerative disease characterised by autonomic dysfunction and/or parkinsonism, cerebellar or pyramidal features [1]. Pathologically, *α*-synuclein-positive glial cytoplasmic inclusions (GCI) and neuronal loss are seen in the substantia nigra, caudate, putamen, globus pallidus, inferior olives, pontine nuclei, and cerebellar Purkinje cells. In the spinal cord, degeneration is evident in the intermediolateral (IML) cell columns and Onuf's nucleus [2, 3].

Autonomic dysfunction presents as lower urinary tract (LUT) dysfunction, erectile dysfunction (ED) and orthostatic hypotension (OH). LUT symptoms present on an average 2.8 years sooner than motor symptoms and may be the initial presenting complaint in nearly 20% of patients [4], preceding OH [5, 6]. Commonly, patients present initially with storage LUT symptoms such as nocturia, urgency and urinary incontinence, and over time voiding difficulties become more apparent [5]. The post-void residual (PVR) volume increases with disease progression, and in a published series the mean PVR in the first year of disease was 71 mL, which increased to 170 mL by the fifth year. Urinary retention requiring catheterisation is rarely reported at disease onset, and increases to 14% over a 5-year period [7].

However, we have observed and we illustrate in this paper a subset of representative patients who initially presented with severe urinary retention requiring catheterisation, bowel and sexual dysfunction, and abnormal anal sphincter electromyography (EMG) which is otherwise characteristic of sacral spinal cord (SSC) lesions, and only subtle neurological signs that would not fulfil the diagnostic criteria of MSA. Subsequently, the disease progressed and all of them developed characteristic signs of MSA leading to clinical diagnosis.

We postulate that at least in a subset of patients, the pathology of MSA can start in the sacral spinal cord and subsequently spread to other areas of the central nervous system.

Table 1 summarises the findings of six cases and a representative case is given below.

**Table 1:** Clinical characteristics of six patients with probable MSA presenting initially in urinary retention, with little neurological signs

Patient	Gender/age	Duration of UR/catheter use (years)	Duration of SD/BD (years)	Duration of diagnosed OH (years)	Interval between UR and onset of neurological signs (years)	Interval between UR and diagnosis of probable MSA (years)	AS EMG	UDS findings—storage phase	UDS findings—voiding phase	PVR (ml)	Type of MSA
1	M 37^a,b^	3/3	5/3	–	7	7	ABN	Open BN	DU	517	MSA-C
2	F 56^b^	2/1	–/1	2	1	3	ABN	SUI, no DO	DU	182	MSA-P
3	M 60^b^	4/3	4/-	2	2	3	N/A	N/A	N/A	200	MSA-C
4	M 48	1/1	1/1	–	1	1	ABN	N/A	N/A	N/A	MSA-P
5	M 60	2/2	9/-	–	2	2	N/A	N/A	DU	300–450	MSA-P
6	F 66^b^	3/3	2/2	Mild	3	5	ABN	DO, ↓C	N/A	150–200	MSA-P

*UR* urinary retention, *SD* sexual dysfunction, *BD* bowel dysfunction, *OH* orthostatic hypotension, *AS* anal sphincter electromyography, *UDS* urodynamics, *PVR* post void residual, *ABN* abnormal, *N/A* not available/not performed, *DO* detrusor overactivity, *DU* detrusor underactivity, *BN* bladder neck, *SUI* stress urinary incontinence, *C* compliance, *MSA-P* parkinsonian phenotype, *MSA-C* cerebellar phenotype

^a^Autopsy confirmed MSA

^b^Deceased

### Patient 1

**Fig. 1:**
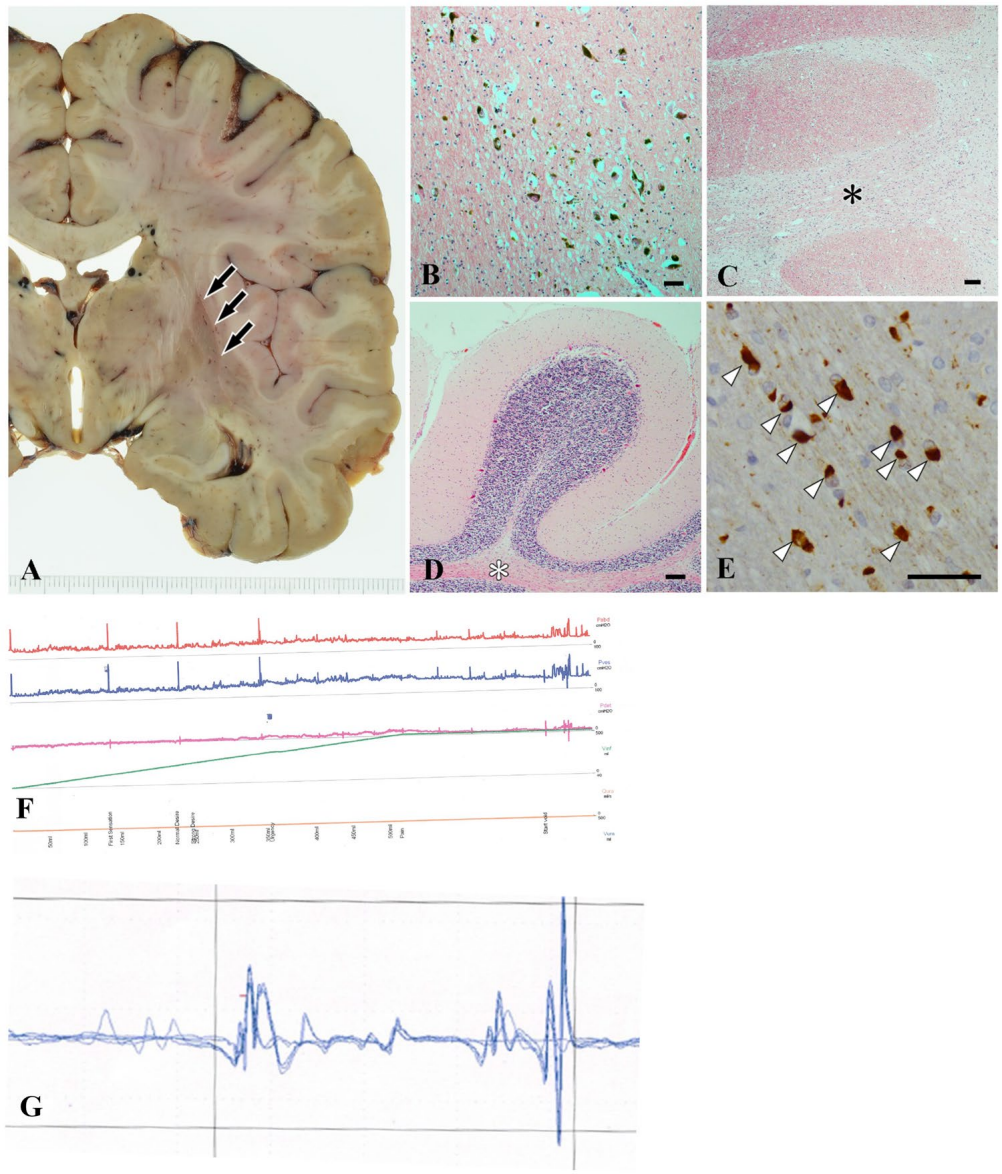
Findings of case 1. **a**–**e** Pathological findings. Coronal section of the left cerebral hemisphere showing atrophy and dark discolouration in the putamen (arrows) (**a**). Moderate loss of dopaminergic neurons in the substantia nigra (**b**). Moderate depletion of neurons with rarefaction of the transvers fibres in the pons (black asterisk) (**c**). Moderate loss of Purkinje cells along with loss of myelinated fibres (white asterisk) (**d**). Glial cytoplasmic inclusions immunopositive for α-synuclein (arrowheads) (**e**). **b**–**d** Haematoxylin–eosin staining, **e** α-synuclein immunohistochemistry. Bars = 50 μm (**b**, **e**); 100 μm (**c**, **d**). **f** Urodynamics findings showing normal compliance, normal bladder capacity, stable detrusor during fill, normal bladder sensation during fill, and acontractile detrusor during the voiding phase. *Pabd* intra-abdominal pressure, *Pves* intravesical pressure, *Pdet* detrusor pressure, *Vinf* infused volume, *Qura* urine flow. **g** Concentric needle EMG of the external anal sphincter. Duration of the recorded motor unit is 38.54 ms, which is prolonged and suggests chronic reinnervation. The mean duration of MUPs during the study was 19 ms (normal < 10 ms) and the EMG was compatible with a diagnosis of multiple system atrophy (gain 0.2 mV/division, sweep speed 10 ms/division)

Patient 1 developed insidious onset voiding difficulties, urinary frequency and urgency, and when assessed 2 years later was found to be in urinary retention. He was experiencing erectile and ejaculatory dysfunction, and constipation. At the time of referral for investigating the cause for urinary retention, he was unable to void naturally and was performing intermittent self-catheterisation four times a day. The neurological examination revealed brisk deep tendon reflexes and no other neurological signs. The blood pressure was stable. MR imaging did not show any significant atrophy in the brain, or compressive lesions of the conus or cauda equina. The results of anal sphincter EMG and urodynamics testing are presented in Fig. 1.

Two years later he developed postural instability, a cerebellar gait, dysphagia and REM sleep behaviour disorder (RBD). Autonomic testing showed evidence for cardiovascular autonomic failure and a diagnosis of probable MSA was made. The following year, he developed dysarthria, bradykinesia, rigidity and worsening cerebellar signs and became wheelchair bound. Repeat brain MRI showed mild parenchymal volume loss within the posterior fossa, mainly affecting the middle cerebellar peduncles, although the cerebellar fissures also appeared marginally more prominent. The pons also appeared to have lost volume, with the impression of more conspicuous midline linear T2-hyperintense signal change. The basal ganglia structures returned normal signal.

His mobility deteriorated and he became bed bound and required a urethral indwelling catheter. He died of complications related to MSA at the age of 43. At autopsy, histological examination confirmed the diagnosis of MSA with neuronal loss predominantly affecting the olivopontocerebellar structures, and to a lesser extent the striatonigral structures, corresponding to the OPCA subtype. There were very frequent glial cytoplasmic inclusions and threads in the pontine base with slightly less frequent deposits in the tegmentum, including the locus coeruleus (Fig. 1).

## Discussion

In this series of six cases, the initial clinical picture was dominated by urinary retention requiring either intermittent or an indwelling catheter, accompanied by bowel and sexual dysfunction with only subtle neurological signs. Patients subsequently developed neurological signs and only at this point met the diagnostic criteria for probable MSA [1]. Brain tissue from patient 1 clearly demonstrated a pattern of neuronal loss and the presence of glial cytoplasmic inclusions and threads consistent with the diagnosis of MSA.

This presentation is in contrast to the more common clinical presentation of urinary retention developing after the onset of other neurological signs, usually after the second year of illness [7]. Urodynamic testing revealed detrusor underactivity which, in the setting of neurological disease, occurs as a result of a lesion of the sacral spinal cord or infrasacrally [8]. In MSA, urinary retention can occur due to detrusor underactivity, which has been reported in 71% of women and 63% of men in a series of patients undergoing urodynamics testing [9]. The prevalence of detrusor underactivity increases with advancing disease, paralleling worsening urinary retention over time [7], and is likely to reflect degeneration in the sacral spinal cord, specifically the parasympathetic IML column.

Urinary retention may also result from involvement of the brainstem such as the pontine micturition centre [10-13]. It was notable that patients in this series had very little neurological symptoms suggesting brainstem dysfunction at the time of presentation with urinary retention. Incomplete bladder emptying in MSA may also occur due to detrusor sphincter dyssynergia, reflecting suprasacral spinal cord involvement [7, 8].

Concentric needle EMG of the anal sphincter revealed abnormal reinnervated motor unit potentials in all the patients who were tested. Nerve fibres innervating the striated anal sphincter are derived from the Onuf’s nucleus in the ventral anterior horn of the sacral spinal cord primarily at the S2 level, but extending between S1 and S3 segments [14]. An abnormal EMG suggests a lesion affecting the sacral somatic motor efferent pathway including the Onuf’s nucleus [15-17].

Co-existent sexual and bowel complaints, and the findings of an abnormal anal sphincter EMG, would suggest a neurological cause for urinary retention with likely localisation at the sacral spinal cord or nerve roots [8, 18]. Orthostatic hypotension was present in some of the patients at an early stage, and a peripheral autonomic disorder such as pure autonomic failure (PAF) may have been considered in the differential diagnosis; however, the anal sphincter EMG is reported to be normal in this condition [15, 19]. However the patients in this series subsequently developed classical extrapyramidal and cerebellar involvement and a diagnosis of probable MSA was made.

We speculate that a subset of patients with MSA present with urinary retention and few neurological signs and the pathology in these cases begins in the sacral cord. The lower spinal cord is particularly involved in MSA and pathological studies reveal neuronal loss and gliosis specifically affecting the IML cell columns, anterior horn cells, pyramidal tracts, and Onuf’s nucleus [20, 21]. Moreover, Schwann cells of the anterior nerves of the sacral cord may show accumulation of phosphorylated α-synuclein [22]. In proteolipid protein-α-synuclein transgenic mice demonstrating MSA-like α-synuclein inclusions and parkinsonian motor deficits, neuronal loss and α-synuclein overexpression have been demonstrated in the Onuf’s nucleus analogue as early as 2 months of life [23]. Neurons of the Onuf’s nucleus share features between somatic and autonomic neurons and is susceptible to neurodegenerative conditions affecting the autonomic nervous system such a MSA [21, 24]. Pre-ganglionic neurons of the IML column of the sacral spinal cord are responsible for the innervation of the detrusor [25] and involvement of this region is likely to be responsible for detrusor failure. Routine MRI sequences of the lower cord are insensitive to identify degenerative changes in the lumbosacral spinal cord; however, high-resolution MR imaging protocols have successfully imaged the lumbosacral enlargement [26] and conus medullaris [27] and changes of grey matter atrophy have been demonstrated in MSA [28].

The transmissibility of α-synuclein has been demonstrated in animal models following inoculation of tissue from MSA patients [29], and the transmission of pathology from sacral cord to other regions of the central nervous system is therefore plausible [30]. Other neurological signs develop with progressive involvement of the brain stem, cerebellum and basal ganglia. Further studies are required to explore this pathological progression from spinal cord to brain, as well as to prospectively chart the clinical progression of patients with suspected MSA who present initially with voiding dysfunction.

In conclusion, a subset of patients with probable MSA may present initially with urinary retention. The findings of detrusor underactivity in urodynamic testing and findings of reinnervation in the anal sphincter EMG suggests possible involvement of the sacral spinal cord. A limitation to the study is that spinal cord tissue was not analysed; however as tissue diagnosis was made at end stage, the inclusion of sacral cord pathological findings would not help support the hypothesis that the disease process actually begans in the spinal cord. Nevertheless, considering the degree of spinal cord involvement in this condition, both brain and spinal cord tissue should be analysed in future pathological studies of MSA. If further studies support the progression of pathology from sacral cord to brain in early MSA, patients presenting with urinary retention and mild neurological features would be an ideal group for experimental trials evaluating neuroprotection in MSA.
